# Beyond birth support: how doulas navigate anti-racism advocacy for refugee and asylum-seeking women in UK maternity care

**DOI:** 10.3389/fpubh.2025.1681812

**Published:** 2025-10-16

**Authors:** Jaime Miller, Sarah Shemery, Gwenetta Curry

**Affiliations:** ^1^Health and Life Sciences, Northumbria University, Newcastle, United Kingdom; ^2^College of Medicine and Veterinary Medicine, The University of Edinburgh, Edinburgh, United Kingdom

**Keywords:** doula, anti-racism, refugee and asylum seekers, maternal health, migration, maternity care, marginalised women

## Abstract

**Background:**

Asylum-seeking and refugee women face significant maternal health disparities in the UK, prompting the emergence of doula and birth companion organisations to provide advocacy and support. This study examines how these organisations train volunteers on advocating for anti-racism in maternity care settings and supporting their clients.

**Methods:**

Semi-structured interviews were conducted with representatives from 12 doula service organisations across the UK, plus two training experts. Interviews explored organisational structure, volunteer recruitment and training, and mechanisms for reporting discriminatory incidents. Data were analysed using grounded theory approaches.

**Results:**

Three key themes emerged: volunteer motivations and expertise varied significantly, with many lacking lived experiences of forced migration; organisations demonstrated inconsistent capacity to document and report racism but saw the need to implement systems; and volunteer demographics often failed to reflect client populations due to structural barriers limiting participation from marginalised communities.

**Conclusion:**

While birth companion organisations provide essential advocacy for asylum-seeking women, systemic barriers limit their full potential. Moving toward paid positions and addressing underlying healthcare racism are necessary for meaningful change. Doula service organisations should be better funded and integrated into the national health system.

## 1 Introduction

Asylum-seeking and refugee (ASR) women in the UK experience profound maternal health disparities that demand urgent policy and practice interventions. International migration continues to increase in scale and complexity, with the Office for National Statistics reporting that 183,309 babies were born to non-UK born women in England and Wales in 2023, representing 30.3% of all live births (1). This demographic shift reflects a 7% increase in births to non-EU born women since 2021—a population with higher representation of asylum seekers and refugees who face compounding vulnerabilities throughout their pregnancy journeys.

These women encounter multifaceted challenges to their physical and psychological wellbeing, stemming from pre-existing health conditions, traumatic migration experiences, and hostile reception conditions in the UK (2–7). The migration journey itself creates cascading negative consequences, including heightened vulnerability to exploitation and sexual and gender-based violence, compounded by challenging settlement conditions (2–4, 8). During the critical perinatal period, these challenges intensify through delayed antenatal care access, inadequate social and cultural support networks, and complex interactions between pre-existing conditions and new stressors (2, 3, 9–11).

The evidence documenting these disparities is stark and unequivocal. Asylum seeker and refugee women experience significantly higher rates of adverse perinatal outcomes (3, 12–14), including elevated neonatal mortality and preterm birth rates (15, 16), increased maternal mortality (3, 13, 14, 17), higher maternal morbidity (3, 13, 14, 17), and greater prevalence of mental health complications (3, 18–20) compared to both other migrant groups and UK-born women. Migrant women face a higher risk of maternal morbidity and mortality than non-migrant women which is partly explained by physical health conditions, previous obstetric experiences and factors related to migration and trauma. The MBRRACE-UK report has brought national attention to pronounced racial variations in maternal deaths, illuminating the complex and intersectional nature of maternal health inequalities facing racialised minorities (21).

These disparities reflect a nearly four-fold and two-fold higher maternal mortality rate for Black and Asian women, respectively, compared to white women in the UK (7, 21). As the pregnant population becomes increasingly diverse, addressing these inequalities has become critical for healthcare equity. At the heart of these disparities lie the systemic racism which “are pervasively and deeply embedded in systems, laws, written or unwritten policies, and entrenched practices and beliefs that produce, condone, and perpetuate widespread unfair treatment and oppression of people of color, with adverse health consequences” (22). Cultural safety, cultural competence, anti-racism, and equity, diversity and inclusion initiatives now feature prominently in healthcare and social service sector agendas (5, 22, 23).

In response to these challenges, paraprofessional roles such as birth companions and doulas have proliferated across the UK, attempting to improve perinatal experiences for vulnerable populations. Doulas are professionally trained support providers who deliver emotional, informational, and psychosocial assistance to individuals throughout the prenatal, birth, and postpartum continuum (24). Rather than replacing medical professionals, doulas work alongside physicians, midwives, and nurses to enhance care provision. Doulas offer personalised physical and emotional support, including guidance, advocacy, and hands-on help to address the nonclinical needs of birthing individuals (24, 25).

Community-based volunteer doulas offer nonclinical support throughout pregnancy and the postpartum period and are increasingly recognised as an evidence-based strategy to promote birth equity. They often deliver comprehensive physical and emotional support during pregnancy, birth, and after delivery, often at little or no cost to clients. Community-based doula organisations are often voluntary and community sector enterprises that support vulnerable and disadvantaged populations (15, 26, 27). However, there is limited clarity around the specific tasks they perform and how their time is allocated across these activities. Crucially, doulas maintain clear boundaries by not engaging in clinical procedures, offering medical guidance, or making clinical interventions (24, 25). Other research describes labour companions (which includes doulas) must also be trustworthy and compassionate. The field encompasses various specialisations, with practitioners often identified by their primary focus area, such as prenatal support, birth attendance, breastfeeding assistance, or postpartum care (28).

These doula service organisations and their volunteers face tremendous strain supporting clients who encounter both micro and macro-aggressive racism and discrimination (23, 29, 30). The implementation of healthcare policies and practices characterised by anti-immigration sentiment and rhetoric that fosters public hostility toward migrant populations compounds the difficulties experienced by birth companions when advocating for their clients (6, 8, 31).

This research examined the landscape of birth companion charity organisations specifically serving asylum seekers, refugees and undocumented migrants (ASR) in the United Kingdom. Through qualitative interviews across these organisations, we investigated: (a) volunteer selection processes, (b) training approaches for supporting clients during racist and discriminatory encounters, and (c) organisational support systems for processing and documenting such incidents. Our inquiry focused particularly on anti-racism, cultural competence and cultural safety training delivery, alongside ongoing support mechanisms as volunteers develop strategies to advocate for vulnerable families.

Recent research demonstrates that patients from Black, Asian and Minority Ethnic (BAME) communities in the UK experience both overt discrimination and anticipated discrimination, requiring advocacy while navigating cultural differences with maternity care providers (8, 23). Systematic reviews reveal predominantly negative experiences among migrant women from racialised minority backgrounds in UK maternity care systems, including communication barriers, discrimination, lack of cultural safety, access challenges, physical discomfort, and discontinuous care (23, 26, 31). Three primary factors negatively impact undocumented migrant women’s maternity experiences: restricted agency, intersecting stressors, and ongoing cycles of precarity (31). The term “racialised minorities” describes Black, African, Asian, Brown, mixed-heritage, and Indigenous populations who experience systemic discrimination based on racial categorisation (22). Understanding gender equality differences and cultural sensitivity is also noted as an important consideration for migrant women in maternity care (9, 23, 32).

Community-based doulas for migrant and refugee women fulfill multiple roles: increasing maternity service capacity, improving health outcomes and experiences for women, and demonstrating implementation and sustainability potential for doula programs (25, 26). Birth companions sharing cultural or linguistic backgrounds play particularly important roles supporting families with limited birth knowledge (26, 28). ASR women face additional challenges in UK maternity care due to both pre-migration trauma and ongoing asylum system stresses, creating hostile birth environments (2, 8, 33). There are three primary outcomes ASR women seek from UK maternity experiences: safety, equal and fair treatment, and future-building opportunities (26, 32). Respectful care, communication and relationships between health care providers and migrant women is enhanced by community-based doulas (25, 26).

Community-based doulas often work with vulnerable populations characterised by low income and minimal support networks (27, 33). They serve as crucial mitigating factors for maternal mortality and other risks facing birthing families, particularly in low-income communities and communities of color (30, 33). Women receiving continuous pregnancy, childbirth and postpartum support demonstrate improved clinical outcomes, including reduced caesarean section rates and enhanced birth experiences (21, 24). This support proves especially vital for refugee and asylum-seeking individuals in the UK, given elevated risks of postnatal depression and psychological challenges including loneliness, social isolation, and pre- and post-migration stress significantly impacting mental wellbeing and physical health (30, 33).

UK organisations demonstrate considerable variation in size, structure, training approaches, recruitment strategies, referral systems, data collection methods, and evaluation processes (15, 29, 34). Some maintain large staff contingents, generate revenue through training provision, and support substantial client populations. Others operate entirely through volunteers without paid staff, often struggling to meet community demands (15, 28). Despite this variation, birth companions have increased across the UK, responding to persistent maternal health disparities and attempting to improve perinatal experiences for vulnerable populations (21, 26, 32).

The emergence of community-based birth companions specifically serving ASR and BAME populations represents a critical intervention attempting to mitigate systemic barriers in maternity care (23, 29, 30). Research from the United States demonstrates that doulas act as advocates and maternal justice leaders, implementing “shifting strategies to address racism in maternal healthcare settings” (29, 30). For doulas who are women of color, motivation for entering this work often centers on “supporting women from the doula’s own racial, ethnic, and cultural community” (30, 35). Research shows that Black doulas provide pathways for Black birthing individuals to navigate systemic racism during perinatal processes, while acknowledging that for Black doula communities to flourish, underlying racism must be eliminated (34, 35). Fair payment models and the high cost of affective labour in the context of racial discrimination are important considerations for doulas (30, 35). Renumerating doulas is noted as potentially strengthening and sustaining doula service providers which may overcome barriers such as retaining volunteers (28).

Organisations such as AMMA have provided birth companion support for ASR communities in Glasgow, Scotland (33). Their influential report describes client experiences characterised by personal trauma histories intersecting with ongoing asylum system stress, often culminating in births within hostile healthcare environments (29, 33). All authors participated in the writing of this report which helped conceptualise this specific research inquiry.

Most birth companion organisations operate through volunteer leadership or volunteer doulas (28, 34). Spiby et al. (34) documented varied doula motivations encompassing both personal factors (confidence building, qualifications, training opportunities, social capital development) and work-related considerations (experience in health and social care settings). Doulas fulfill diverse roles spanning antepartum through postnatal periods, including navigation of complex health and social care systems and assistance addressing socio-economic disparities (26, 34). The foundation of doula work rests on peer support, grounded in developing “socially meaningful relationships of trust” (28) with potential to improve physical health outcomes through enhanced service access, improved mental and emotional health through reduced social isolation, and decreased stress and anxiety (28, 36). This support proves particularly meaningful for women from racialised and disadvantaged backgrounds (23, 30, 35).

## 2 Materials and methods

### 2.1 Study design

This qualitative, exploratory study sought rich descriptions of racism and discrimination experiences encountered by birth companions and their clients, alongside training received by companions to advocate effectively in hostile settings (36, 37). Qualitative research methods prove particularly valuable when studying marginalised groups and understanding experiential nuances (37). This approach enabled contextual understanding of cultural safety and advocacy training approaches within broader organisational and community contexts.

### 2.2 Study setting and sampling

The study encompassed the United Kingdom excluding the Republic of Ireland. Initial mapping exercises identified eighteen organisations primarily serving refugee, asylum-seeking, and undocumented migrant populations or making specific mention of these communities on organisational websites. Some organisations solely support ASR women, while others primarily serve ASR or other migrant women due to their location. Most organisations operated in England, with one each in Wales and Scotland. No doula or birth companion organisations were found in Northern Ireland. Twelve organisations participated in interviews, alongside two individual experts who conducted training for these organisations, providing additional context on specialised training development. All identified organisations were contacted, with additional organisations identified and contacted as interviews progressed.

### 2.3 Participants

Participants primarily held senior leadership roles within their organisations and possessed familiarity with birth companion training, organisational structure, and functioning. The participants were all female identifying and were from a range of British-born white women to British-born BAME women. Some of the participants were also migrants.

### 2.4 Data collection

Northumbria University Newcastle provided ethical approval in winter 2025 (Miller 2024-8315-9790), with interviews commencing shortly thereafter. Participants received comprehensive packages including project debriefs, consent forms, and interview questions for pre-interview review. There was no pilot testing of the interview questions. Semi-structured interviews explored four main themes: organisational structure, client populations served, cultural safety training, and organisational aspirations. Interviews lasted 45–60 min and were conducted via MS Teams. There were no field notes collected. There were no non-participants in the interview and no repeat interviews.

All interviews were transcribed verbatim and returned to participants for approval, with three-week windows provided for transcript modifications. Data collection continued until saturation was achieved, identified through lack of new information and perspectives at the tenth interview (37). Once transcript verification was completed, coding structures were established utilising grounded theory principles and constant comparative analysis (36, 37), allowing for sophisticated interpretation through systematic theme comparison and iterative refinement. The research team comprised two social science academics and one doctoral candidate, all student and work visa migrants from North America. One of the authors is the child of refugee parents. The authors reflexivity to consider their own experiences of migration when considering the experiences of the participants. Two authors (JM and SS) conducted the interviews either individually or as a team. They are both experienced qualitative interviewers and work with doula organisations and had familiarity with their structure, training and service delivery operations. SS also leads anti-racism training for a doula organisation and had some insider perspectives on training formats.

All three authors independently developed coding frameworks based on the transcripts, with the lead author compiling individual frameworks into a common framework agreed upon by all team members (36, 37). Each transcript was individually coded using selective approaches analysing frequently coded themes most relevant to original research aims. The interviews were organised and analysed using Microsoft Excel pivot tables to categorise coded responses, calculate frequency distributions of thematic categories, and examine cross-tabulations between variables to identify emerging patterns and relationships within the dataset. Through multiple meetings, the team compared individually coded transcripts and adjusted the common framework as needed, with all differences discussed and debated. Following analysis completion, dominant themes were identified and manuscript preparation commenced. A brief infographic and manuscript explanation were sent to participants as member-checking mechanisms ensuring accurate conversation interpretation (37).

## 3 Results

Several key themes emerged from interviews, including data collection practices, anti-racism training approaches, trauma-informed care support, volunteer motivations and expertise, volunteer-client demographic alignment, and mechanisms for reporting racist and discriminatory experiences. This paper examines three intersecting themes in detail: (a) volunteer motivations and expertise, (b) volunteers’ capacity to record and report racism incidents, and (c) volunteer representation of client populations.

### 3.1 Volunteer motivations and expertise

Organisations primarily relied on volunteers as birth companions and doulas, with some receiving nominal honoraria, expense reimbursements for travel, and occasional meals. All volunteers completed mandatory in-house training or intake processes with their organisations, with several receiving additional training from other doula organisations. Volunteer motivations varied considerably including wanting experience working in the field to wanting to connect with other volunteers. Many volunteers had previously worked in maternity care as midwives or nurses or were midwives in training.

Organisations acknowledged that volunteer work’s unpaid nature created limitations regarding who could engage in unpaid labor and the privileges associated with this approach. For volunteers with maternity care background, both benefits and disadvantages emerged. The National Health Service (NHS) maternity care understanding and general systems navigation represented important benefits for volunteers with health and social care backgrounds. However, interviews revealed significant blind spots regarding issues clients and families faced in hostile environments. Failing to recognize neglectful care as racism for these populations exemplified such blind spots. As one participant stated: “I’ve been feeling this for a while anyway, but a lot of our doulas are white, so they are necessarily not going to recognise the nuances that someone of colour is going to be kind of experiencing.” One participant described requiring doulas to possess extensive knowledge either as trained doulas or midwives, noting this requirement’s impact on volunteer backgrounds: “that ends up limiting the amount of representation that we can have within that group of people”.

### 3.2 Volunteer capacity to record and report racism incidents

Doula organisations demonstrated varying mechanisms for reporting racist incidents or poor client treatment. Some organisations and volunteers were well-versed in incident reporting procedures and protocols. Well-established organisations expressed confidence in addressing situations as they arose. One participant described challenges around translation services:

“Endless, you know, definitely, and we have witnessed that as well, you know, as doulas, particularly with refugees who do not speak English…a lot of the refugee clients that the project has worked with have been told they are having a caesarean section, and we know that the hospital were doing this because they just could not be asked to provide translators and care for, you know, a birthing person that could not speak in English…racism in maternity care is, you know, rife, absolutely rife. We witness it, call it out. Change the midwife.”

Participants described how doula presence provides additional witness perspectives for potential client mistreatment. One participant noted that people may not realize they are being racist or treating someone differently despite evident body language and micro-aggressions. However, doula presence itself creates impact:

“I do not think that when I’m saying that they sit up, you know, that they are consciously going, okay, there’s a doula here. ‘I need to be more careful.’ I think it subconsciously happens… and I actually think that’s more scary… because there absolutely is definitely a change when there’s a doula in the room.”

Participants also described maternal fears regarding reporting poor treatment and potential life consequences. For many women already embedded in multiple systems, ensuring smooth progression through asylum-seeking processes remained paramount. Reporting healthcare racism and poor treatment often appeared to jeopardize approval chances for remaining in the country. Participants also describe how ASR women are fearful of potential retaliation/punishment or impacts on their asylum application if they do complain about poor care. One participant described a specific situation:

“The doula was Bangladeshi, the mum was Pakistani, and the anaesthetist in the room, who normally we have consistently fantastic feedback from… there was really poor feedback about an anaesthetist who was speaking to the mother in Urdu Punjabi very disrespectfully… we sent that through quite close to when it had happened… it was immediately responded to and asked if we could have more details… it was taken to the head Anaesthetist… but the mum was so scared and worried that it was going to come back negatively on her and impact and get her into trouble with social care… even though she said yes and consented… the following week, she was so worried.”

Volunteers and doula service organisations are aware of some of these complexities and prioritise consent from clients before moving into any formal complaint process that may impact the woman’s reputation in the community alongside any perceived administrative impacts with the asylum-seeking process.

### 3.3 Volunteer representation of client populations

Variations existed regarding volunteer doula reflection of client populations. Some charities explicitly only bring in volunteers with lived experience or from a certain background to ensure they could relate to/effectively support clients. One participant stated:

“They’re all local. There is a difference, probably, in demographical makeup of the volunteer group compared to the community… the clients we serve. There is a mix, and there is a reflection to a good degree, but there are more white British doulas, probably percentage wise than would be reflected in our client group.”

Organisations described increasingly recruiting doulas and birth companions from racialised minority backgrounds to reflect served communities. As one participant noted:

“Because it’s London and it’s a very multicultural place, a lot of the doulas do speak other languages, and they do have their own experience of having either come from somewhere else or being part of other communities as well, but very few of them are from the asylum community.”

Recruiting paid staff and doulas from these communities has become increasingly prioritized and recognized as essential for effective advocacy. As one participant described:

“We do try and encourage applications from volunteers with a lived experience…encouraging volunteers from global majority backgrounds with this particular cohort. And there’s one volunteer from a global majority background. There’s nobody with a lived experience of the asylum system. But it is something when we are advertising our volunteer opportunities that we encourage applications from that group.”

Participants described recruitment challenges, often stemming from unpaid work privileges. One participant emphasized English fluency requirements for effective advocacy “in a birth space with hospital jargon and interpreters.” Another noted volunteer scheduling flexibility requirements: “you need to have childcare and flexibility. A lot of our volunteers will be middle class white women.”

Regarding recruiting volunteers from served communities, one participant observed: “if we want doulas who come from marginalized communities… and we want them to work, we need to put them in a position that can be reimbursed, at least for their time.”

Another participant described modified recruitment approaches, employing paid staff who were from the ethnic and cultural communities to visit gathering spaces like coffee shops and encourage women to volunteer: “we have done a lot of work to make our recruitment process and our training more accessible. Every training block is more diverse than the one before.”

## 4 Discussion

This research identified that doulas volunteering for organisations primarily serving refugee and asylum seekers bring diverse expertise, motivations and backgrounds to their roles (29, 30, 34). While serving in these positions, doulas frequently witness clients experiencing poor treatment in hostile maternity environments (6, 23, 31). Doula organisations vary considerably in equipping volunteers with reporting and response procedures, with some escalating concerns to senior staff while others leave complaint processes entirely to clients and families (6, 7). For this vulnerable population of birthing women, doulas represent essential experiential components (25, 26, 28).

The increase in UK doula services for ASR women connects to several factors. Increasing migrant influx seeking refuge, as reported by ONS (1), has expanded demand for services specifically supporting ASR women’s needs. Doula services directly address family network losses and social and cultural support gaps (2, 3, 9–11, 28). ASR-supporting doula services have proliferated alongside MBRRACE-UK work documenting substantial maternal mortality rate differentials for Black and Asian women (21). The surge in far-right anti-immigration movements in the UK, notably following the summer 2024 Southport stabbing incident affecting young girls attending dance class, with three tragic deaths and subsequent protests, has further exacerbated hostilities experienced by migrant women, increasing doula work importance (8, 22, 31).

### 4.1 Challenges with volunteer doula advocacy

Volunteer doula work environments are often shaped by negative experiences of served women (2, 23, 31). Participants described language and communication challenges, poor care access, and cultural safety deficits like those reported by Obionu et al. (23) meaning doulas provide not only pregnancy support but also navigate all care barriers clients face (6, 26, 32). As Spiby et al. (34) reported, volunteer doulas possess both personal and work-related motivations yet now occupy front-facing roles witnessing and reporting experiences of women in hostile maternity environments. Because doula work remains voluntary, only those in economic and social positions enabling unpaid work typically become volunteer doulas (30, 34). Consequently, many volunteers lack direct ASR experience, although volunteer diversity is increasing (30, 35). Inability to understand poor treatment nuances due to limited lived experience could further isolate ASR women (23, 35). Since doula work centers on building socially meaningful relationships (28) and compassionate and trustworthy care (28), training doulas to understand ASR healthcare system complexities better equips them for effective advocacy (28). The doulas also take on additional affective labour as they navigate racially hostile environments while advocating for their ASR clients (30).

### 4.2 Doula volunteer capacity to record and report racism

Participants and doula service organisations undoubtedly operate at the forefront of witnessing, processing and sometimes reporting maternity care racism (25, 26). Despite increasing cultural safety focus, clear variations persist between white, British-born maternal outcomes and racialised minority ethnic group outcomes that doula organisations witness firsthand (21–23). Due to ASR women’s precarious lives served by doula organisations, incident reporting challenges arise from repercussion fears and perceived asylum claim impacts (2, 6, 8, 31).

Participants described doulas serving as witnesses, potentially reducing hostile and poor treatment of pregnant women by maternity staff (29, 30). Doula service organisations are embedded into formal NHS structures in varying ways (6, 7). Some maintained direct communication lines with hospital maternity leadership, enabling swift concern raising and addressing. Others relied on NHS website complaint systems (7).

Participants also described internal systems for logging poor treatment including racism. Some organisations utilised software programs like Charity Log, while others maintained incident spreadsheets. No organisations felt their current systems adequately recorded and documented incidents, with all considering this an aspiration.

### 4.3 Diversity and lived experience recruitment

Doula organisations aspired to maintain doula populations reflecting served client populations (22, 30, 35). Various recruitment efforts were described for engaging communities in volunteer doula workforce recruitment. Primary barriers included unpaid work nature and additional volunteering obstacles such as childcare and transportation difficulties limiting racialised minority community recruitment (30, 34).

Some organisations employed staff from racialised minority backgrounds, including former clients turned volunteers, to visit spaces frequented by women from various cultural and community backgrounds (22, 35). Diverse doula populations serving diverse client populations serve multiple purposes, providing additional familiarity layers around culture, language and common practices promoting “socially meaningful relationships” (28). As Khaw et al. (26) assert, doulas sharing language or culture play important roles when birth knowledge is limited, making these UK doula organisation recruitment shifts toward diverse backgrounds integral. Understanding other gender-based differences and cultural sensitivities (9, 23, 32) is another layer of nuanced required by doulas working in migrant and ASR communities.

Doulas reflecting client populations in culture, language and ethnicity provide critical roles for ASR women facing racist maternity treatment (30, 35). As Thomas et al. (35) note, despite facing institutionalised, interpersonal, and internalised forms of racism, Black community-based doulas provide avenues for Black birthing individuals to navigate systemic racism experienced during the perinatal process, though these forms of racism need to be addressed for Black community doulas to flourish (22, 35). Participants described this leadership and advocacy type that doulas and organisations initiate and consider it a key part of their mandates (29, 30).

Despite having doulas from the same ethnic, cultural and linguistic groups as clients, and well-defined racism reporting policies and procedures, pregnant ASR poor treatment remains unacceptable (2). Poor perinatal outcome root causes for this population are intersectional and complex (2, 20, 21). Negative impacts are exacerbated when ASR women receive poor treatment and experience racism in maternity care (21, 23, 31). While doulas provide companionship, comfort, support, guidance and advocacy, systemic injustices shaping women’s maternity care interfaces remain core issues (7, 22, 31).

## 5 Conclusion

This research illuminates the critical yet complex role of doula organisations in supporting asylum-seeking and refugee women through hostile maternity care environments in the UK (8, 23, 29, 31). The study reveals significant challenges in volunteer recruitment, training, and advocacy effectiveness, particularly highlighting how the unpaid nature of doula work systematically limits diversity and lived experience representation among volunteers (29, 30, 34). While these organisations demonstrate varying capacities to document and report incidents of racism and discrimination, they consistently serve as essential witnesses and advocates in settings where ASR women face disproportionately poor treatment (6, 23, 29, 31).

Our findings underscore a fundamental tension: despite well-intentioned efforts to match doulas with clients from similar cultural and linguistic backgrounds, systemic barriers including economic privilege requirements for unpaid work continue to limit the representativeness of volunteer cohorts (22, 30, 34). This creates a situation where those most capable of understanding and responding to the nuanced experiences of racism and discrimination—individuals with lived experience of the asylum system—are systematically excluded from volunteer roles due to economic necessity (30, 34, 35).

The implications of this research extend beyond individual organisational practices to broader questions of maternal health equity and systemic racism within UK healthcare (7, 21, 22). While provide invaluable support in mitigating some barriers to care, their presence alone cannot address the root causes of maternal health disparities faced by ASR women (2, 23, 31). The study calls for fundamental changes in how these services are funded and structured, moving toward paid positions that would enable greater participation from community members with lived experience of the asylum system (30, 34).

Furthermore, our research demonstrates that while doula organisations serve as crucial stopgap measures in an inequitable system, achieving true maternal health equity for ASR women requires addressing the underlying hostile environment policies and embedded racism within maternity care settings that necessitate such advocacy in the first place (6–8, 22, 31). Only through systemic transformation—rather than relying solely on volunteer advocacy to ameliorate institutional failures—can meaningful progress be made toward ensuring safe, respectful, and equitable maternity care for all women, regardless of their immigration status or country of origin.

## Data Availability

Owing to the sensitive nature of the research, interviewees did not consent to the retention or sharing of their data. Further inquiries can be directed to the corresponding author.
